# Supplementary material for: Multivariate Analysis of Biochemical Properties Reveals Diversity among Yardlong Beans of Different Origins
Source: Antioxidants (Basel). 2024 Apr 14;13(4):463. doi: 10.3390/antiox13040463 (PMC11047418; doi:10.3390/antiox13040463)
Supplement: Supplementary file 1 [file antioxidants-13-00463-s001.zip › antioxidants-2948972-supplementary.pdf]

Supplementary material

Multivariate Analysis of Biochemical Properties Reveals Diversity among Yardlong beans of Different Origins

Yu-Mi Choi [1], Hyemyeong Yoon [1], Myoung-Jae Shin [1], Sukyeung Lee [2], Jungyoon Yi [1], Young-ah Jeon [1], Xiaohan Wang [1] and Kebede Taye Desta [1,*]

[1] National Agrobiodiversity Center, National Institute of Agricultural Sciences, Rural Development Administration, Jeonju 54874, Republic of Korea.

[2] International Technology Cooperation Center, Technology Cooperation Bureau, Rural Development Administration, Jeonju 54875, Republic of Korea.

* Correspondence: kebedetdesta@korea.kr (K.T.D)

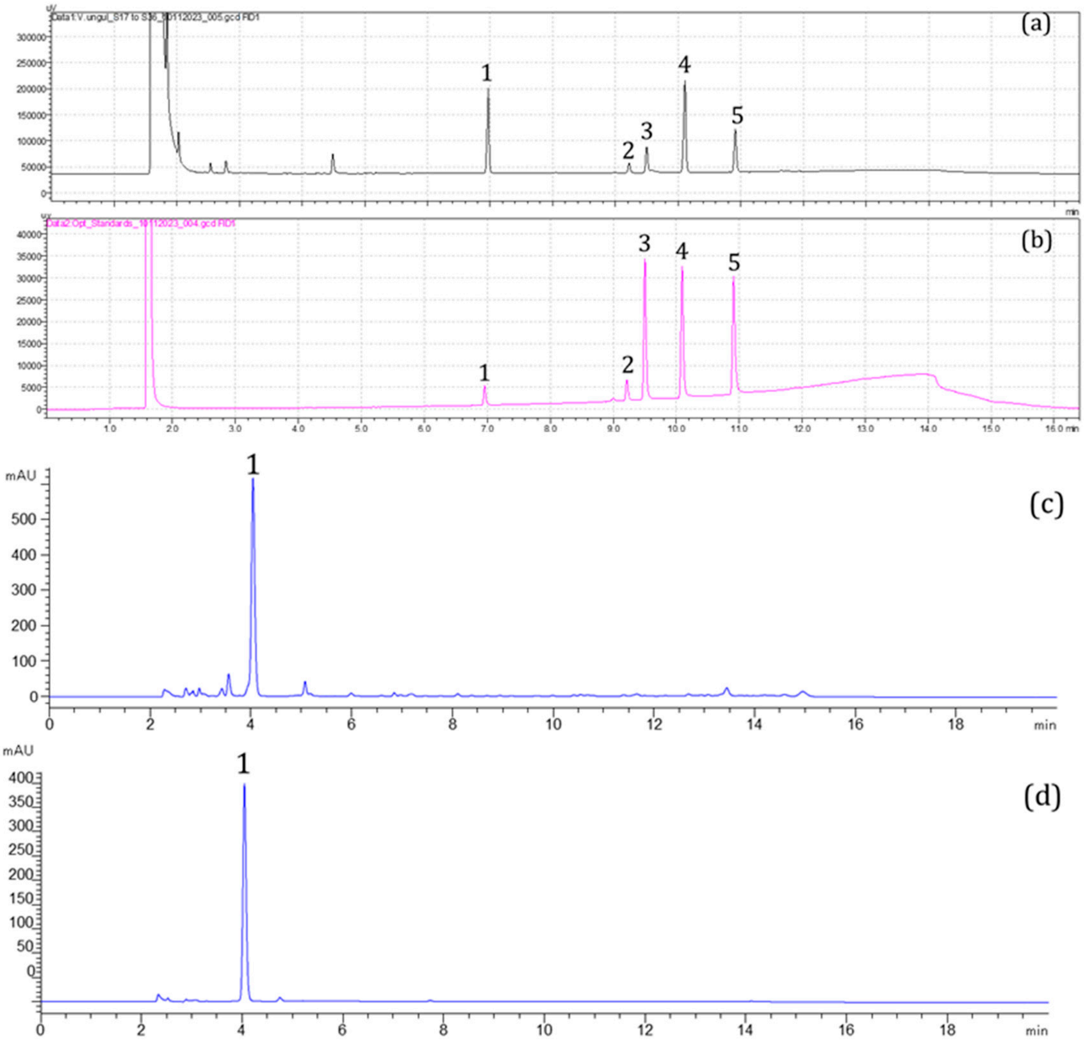

Figure S1. GC-chromatograms of a representative sample (a) and fatty acid standards (b), and LC-chromatograms of a representative sample (c) and *L*-ascorbic acid standard (d). Peak assignments: GC-chromatograms: Palmitic acid (1), Stearic acid (2), Oleic acid (3), Linoleic acid (4), Linolenic acid (5); LC: Chromatograms: Ascorbic acid (1).

Table S1. Frequencies and distribution of qualitative agronomical traits across 35 yardlong beans grown in Korea.

Variable	Categories	*f*	Rel. *f* (%)
Growth habit	Indeterminate	32	91.43
	Determinate	3	8.57
Flower color	Light purple	32	91.43
	White	3	8.57
Pod color	Pale tan	16	45.71
	Mixed	9	25.71
	Dark brown	7	20.00
	Light brown	3	8.57
Pod curvature	Mixed	14	40.00
	Coiled	18	51.43
	Curved	1	2.86
	Slightly curved	2	5.71
Pod pubescence	No hair	35	100.00
Seed shape	Kidney shaped	35	100.00
Seed coat color	Black	3	8.57
	Brown	25	71.43
	Other	6	17.14
	Tan	1	2.86
Seed coat lust	Dull	35	100.00
Hilum color	Eye absent	35	100.00

Table S2. Statistics of quantitative agronomical traits across 35 yardlong beans grown in Korea.

Name	Introduction/temporary Number	Origin	DF (Days)	DFM (Days)	DM (Days)	PL (cm)	SPP (n)	HSW (g)
San chi lu	217325	China	48	25	73	71.40 a	16.00 a-f	18.00 ef
901 Qingjo jiangdou	250133	China	53	19	72	59.60 b-g	14.40 b-i	14.37 mn
Qing jiang 2006	260948	China	54	18	72	65.60 ab	17.20 a-e	14.13 n
Gaotian jinguan	302034	China	54	18	72	61.00 b-f	13.80 d-i	14.53 m
Gyeonggiyonginsujib	K035303	Korea	56	21	77	43.20 lmn	16.00 a-f	16.63 j
Gangwonpyeongchang-2003-4	K037279	Korea	56	17	73	46.40 j-m	16.40 a-f	17.07 i
Gangwonpyeongchang-2003-17	K037292	Korea	57	16	73	42.80 lmn	19.00 a	17.73 fg
Hang xin taikong wu jia dou	K052084	China	47	24	71	32.10 op	15.00 a-i	14.37 mn
Techang 908 jiangdou	K052086	China	54	18	72	57.40 c-g	17.40 a-d	13.13 p
Taiwan chunqiu hong	K052109	China	56	28	84	52.80 g-k	15.40 a-h	17.63 gh
Techang 902 jiangdou	K052119	China	50	22	72	58.20 c-g	18.20 abc	16.03 k
Tai Htaung Pe Ni	K127002	Myanmar	57	27	84	54.40 f-i	15.20 a-h	18.93 b
THA-JSH-2008-81021	K146060	Thailand	59	29	88	49.60 h-l	15.80 a-g	22.23 a
Tanjingeun	K161964	Korea	53	21	74	30.60 p	13.00 f-i	8.10 s
Jasaekginggori	K162054	Korea	57	17	74	47.40 i-m	18.00 abc	16.27 k
Gatggeungong	K163690	Korea	57	17	74	46.00 klm	18.00 abc	17.37 hi
MMR-JYH-2010-90	K166990	Myanmar	59	28	87	59.80 b-g	11.60 hi	15.27 l
Sung 99	K168155	Myanmar	54	15	69	37.40 no	14.40 b-i	12.90 pq
Guamian hong jiangdou	K170009	China	57	22	79	53.40 g-j	14.20 c-i	18.13 de
Qiu jiang 512	K170010	China	50	24	74	43.20 lmn	13.20 e-i	19.17 b
Chungbuk Geosan 2011-18	K171518	Korea	56	18	74	43.40 lmn	12.00 ghi	16.13 k
Chungbuk Geosan 2011-245	K171745	Korea	56	18	74	42.20 lmn	16.20 a-f	16.67 j
901 Zaoshu jiangdou	K176603	China	54	19	73	63.80 bcd	17.60 a-d	14.17 n

Techang jinqili	K223095	China	50	22	72	57.40 c-g	17.20 a-e	13.53 o
Nan 1 Variety	K243771	Thailand	56	23	79	55.20 e-h	11.20 i	18.47 c
Hei mei 1	K251318	China	54	18	72	62.00 b-e	16.80 a-f	14.33 mn
Chunqiu hong jiangdou	K251346	China	57	22	79	55.40 e-h	14.60 b-i	18.33 cd
Man di hong wu jia dou	K251347	China	54	17	71	23.80 q	16.20 a-f	12.23 r
Tichun zhi jiang28-2	K251348	China	50	21	71	57.00 d-g	16.80 a-f	17.27 i
Te xuan zhang tang wang	K251349	China	54	18	72	57.70 c-g	18.40 ab	17.77 fg
Hei mei huang zi wang	K253775	China	50	27	77	64.60 bc	18.20 abc	13.53 o
Yard long Bean 287/2556	K253844	Thailand	54	25	79	28.60 pq	17.00 a-f	12.67 q
SD 3135	K255113	Korea	59	22	81	42.60 lmn	16.20 a-f	17.80 fg
KSL 170256	K267332	Korea	57	20	77	42.00 mn	16.20 a-f	17.17 i
Gyeongnamhabcheon-2019-2	K272494	Korea	59	23	82	48.00 i-m	15.40 a-h	13.07 p
Range (Min- Max)			47.00-59.00	15.00-29.00	69.00-88.00	23.80-71.40	11.20-19.00	8.10-22.23
Mean			54.51	21.11	75.63	50.17	15.78	15.86
CV (%)			5.76	17.78	6.32	22.21	12.38	16.50

DF: Days to flowering, DFM: Days from flowering to maturity, DM: Days to maturity, HSW: One hundred seeds weight, PL: Pod length, SPP: seeds per pod. Different letters in a column represent significantly different mean values ($p < 0.05$).

Table S3. Factor loadings and contribution of variables to the variance observed among the yardlong bean accessions in the principal component analysis

	PC1		PC2		PC3		PC4		PC5		PC6		PC7	
**Variable**	**FL**	**%**	**FL**	**%**	**FL**	**%**	**FL**	**%**	**FL**	**%**	**FL**	**%**	**FL**	**%**
DF	0.58	4.21	-0.52	6.69	0.09	0.30	-0.08	0.24	0.02	0.01	0.31	8.36	-0.36	12.97
DFM	0.36	1.65	0.24	1.40	0.10	0.33	-0.40	6.34	0.60	17.20	0.18	2.77	0.27	7.22
DM	0.66	5.55	-0.16	0.59	0.14	0.65	-0.36	5.30	0.48	11.10	0.34	10.27	-0.03	0.06
PL	-0.36	1.59	0.18	0.77	0.51	8.70	0.09	0.35	0.57	15.65	0.05	0.24	-0.13	1.68
SPP	-0.32	1.28	0.15	0.53	0.32	3.57	0.24	2.35	-0.37	6.59	0.33	9.65	0.04	0.19
HSW	0.25	0.77	-0.10	0.26	0.72	17.76	-0.51	10.66	0.06	0.19	-0.22	4.07	0.04	0.14
TP	0.48	2.89	-0.30	2.24	0.40	5.40	-0.07	0.22	0.38	6.93	-0.25	5.63	0.37	13.32
TF	-0.16	0.33	0.62	9.20	0.37	4.61	0.01	0.00	-0.09	0.39	0.08	0.52	-0.17	2.90
CFC	0.02	0.01	0.24	1.38	-0.42	5.96	0.62	15.87	0.53	13.59	0.02	0.03	-0.02	0.05
DFC	0.25	0.79	0.15	0.57	-0.24	1.88	0.60	14.71	0.16	1.29	-0.09	0.66	0.54	29.42
Vit C	0.02	0.01	-0.21	1.04	0.05	0.09	-0.10	0.38	-0.37	6.36	0.67	39.53	0.47	21.47
TPC	0.63	5.04	0.66	10.71	-0.07	0.17	0.05	0.12	-0.08	0.27	0.16	2.26	0.03	0.07
DPPH	0.73	6.73	0.54	7.08	0.17	0.97	0.11	0.46	-0.25	3.06	-0.05	0.19	-0.03	0.10
ABTS	0.81	8.30	0.47	5.37	-0.01	0.01	-0.03	0.04	-0.18	1.61	0.05	0.22	-0.03	0.07
RP	0.82	8.46	0.47	5.42	0.08	0.21	0.03	0.03	-0.25	3.02	0.00	0.00	0.02	0.03
TTC	0.85	9.03	0.25	1.51	0.30	3.08	-0.11	0.48	0.12	0.67	0.02	0.05	-0.04	0.17
TSC	0.75	7.06	0.36	3.09	0.01	0.01	0.09	0.31	-0.19	1.64	-0.29	7.27	0.08	0.71
PA	0.64	5.22	-0.64	9.97	0.21	1.43	0.17	1.18	-0.21	2.15	-0.12	1.23	0.03	0.10
SA	-0.49	3.03	0.01	0.00	0.33	3.61	0.61	14.91	0.30	4.36	0.25	5.56	-0.11	1.12
OA	-0.79	7.87	0.50	6.01	0.26	2.29	-0.17	1.16	-0.04	0.10	-0.04	0.12	0.10	0.92
LA	0.82	8.42	0.17	0.67	-0.22	1.71	0.24	2.29	0.19	1.70	0.09	0.78	-0.22	4.90
LLA	0.00	0.00	-0.40	3.87	-0.68	15.46	-0.46	8.57	-0.01	0.01	-0.01	0.01	0.09	0.78
TSFA	0.48	2.91	-0.66	10.61	0.34	3.87	0.41	6.77	-0.10	0.52	-0.03	0.06	-0.01	0.01
TUFA	-0.48	2.91	0.66	10.61	-0.34	3.87	-0.41	6.77	0.10	0.52	0.03	0.06	0.01	0.01

PUFA	0.69	5.96	-0.13	0.41	-0.65	14.09	-0.11	0.50	0.15	1.07	0.07	0.46	-0.13	1.59
Eigenvalue	7.92		4.12		2.96		2.46		2.10		1.15		1.01	
Variability (%)	31.66		16.48		11.83		9.85		8.40		4.59		4.03	
Cumulative (%)	31.66		48.14		59.97		69.82		78.22		82.81		86.84	

ABTS: $ABTS^{\bullet +}$ scavenging activity, C: Cultivar, CFC: crude fiber content, CHN, China, DF: Days to flowering, DFC: Dietary fiber content, DFM: Days from flowering to maturity, DM: Days to maturity, DPPH: $DPPH^{\bullet}$ scavenging activity, HSW: One-hundred seeds weight, KOR: Korea, L: Landrace, LA: Linoleic acid, LLA: Linolenic acid, MMR: Myanmar, OA: Oleic acid, PA: Palmitic acid, PL: Pod length, PUAF: Total polyunsaturated fatty acid, RP: Reducing power, SA: Stearic acid, SPP; number of seeds per pod, TF: Total fat, THA: Thailand, TP: Total protein, TSFA: Total saturated fatty acid, TUFA: Total unsaturated fatty acid, TPC: Total phenolic content, TSC: Total saponin content, TTC: Total tannin content, Vit C: Vitamin C.
